# Book review of "The estrogen elixir: A history of hormone replacement therapy in America" by Elizabeth Siegel Watkins

**DOI:** 10.1186/1747-5341-3-1

**Published:** 2008-01-02

**Authors:** Carlos Sonnenschein

**Affiliations:** 1 Department of Anatomy and Cellular Biology, Tufts University School of Medicine, Boston, MA 02111, USA

## Abstract

"The Estrogen elixir: A history of hormone replacement therapy in America" by Elizabeth Siegel Watkins is a thoroughly documented cautionary tale of the information and advice offered to women in the perimenopausal period of their life, and the consequences of exposure to sexual hormones on their health and wellbeing.

According to Webster's dictionary, an elixir is a substance sought by alchemists for converting base metals into gold. Like so many other Elixirs peddled over the centuries, there are no such things... despite the eagerness of many to own or benefit from them. The uselessness of elixirs could have suggested *a priori *the needless pursuit of reading *The Estrogen Elixir: A History of Hormone Replacement Therapy in America*, a book by Elizabeth Siegel Watkins, an academic historian who takes 351 pages to tell us the who, the when and the why of this attempt to sell this "new" elixir to millions of women reaching the age at which they approach, go through and pass menopause. The subject is of great public interest because it extends beyond the specifics of the painful process of (mis)representing medical evidence to what it says about a society that considers itself literate, affluent and socially aware.

Professor Siegel Watkins presents a detailed account of the historical record of the subject. The action takes place in a very peculiar society, in a well defined environment, during most of the second half of the XXth century. The place is, of course, the United States of America. A close to cynical rekindling of the argument could be summarized as follows: first, it was necessary to raise the consciousness of people (women, in this case) who may not have noticed until the mid XXth century a need to resolve a non-life threatening problem such as the inconveniences of menopause; second, to propose a "solution" (in this case, an elixir to neutralize the bad sides of menopause) and "create" a market for its commercialization; and finally, to offer to "those in need" (in this case, women in their 40s and older) who could afford to pay for an elixir, all the medical and social justifications to preserve a certainty of safety, or at least, the idea of the harmlessness of this particular elixir. The medical-social issues dealt with in the book cover the last six decades.

It may not be entirely fair to place the protagonists of this book in the two traditional camps of good and evil. Notwithstanding, given the capitalistic socioeconomic model that our westernized society at large has adopted with gusto during the last century, it would be justifiable to identify the drug companies as the perceived evil trying to "add value to the holdings of their shareholders." Consumers could be the innocent targets of the evil doers. However, as in all historical accounts involving humans, there are the virtuous, the marginally virtuous, the marginally corrupt and the irretrievably corrupt. This being a scientifically, socially and medically centered story means that all of the above mentioned characters fill the places where scientists, "activists" and doctors operate.

And, of course, life is more complex than any simplistic caricature. As in all social intercourse, the exchanges of goods among the parties could be negotiated intelligently and "creatively" for the benefit of all participants or may, instead, lead to conflicts that cannot be resolved amicably. Examples of conflicts generated by other widely used consumer products indicate that the Hormone Replacement Therapy (HRT) story became an angry and mischievous one.

Feminism in its broader sense has influenced how women prefer or demand to be treated by, among others, their physicians and by Big Pharma. These influences affected to a significant extent how the dialogue between women and industry proceeded. But, in the end, the results of large epidemiological studies decided who was right about the benefits of the medicalization of menopause and which were the perils of the indiscriminate use of HRT.

As a historian, Professor Siegel Watkins presents a scholarly account of the issues and the positions the participants have assumed and defended in this conflict. Simultaneously, it is not difficult to define on whose camp she would like to be counted on. In my opinion, there is nothing wrong with this "fact of life." After all, the social context in which we all live requires that scholars lay down "the facts" and make us, the readers, see the reasons under which the narrative justifies siding with one or another position. For instance, it is of interest to know that physicians and scientists at large involved in the discovery of the estrogenic drugs in the 1930s were less interested in personal gains than their colleagues after World War II. One may conclude (surprise!) that the profit motive in public relations became exacerbated in the post-WWII era.

The author judiciously indicates where the faults lie and who should be blamed for each position taken. It is better to face the uncertainty with the eyes well open rather than to blindly fall into desperation and accept risks that may become serious as age increases. This historical book should be read by people who have been actively battling from one or another side of the story, by women who sooner or later will have to face the decision to take up medication (HRT) in order to resolve the few and slight or the many and severe discomforts of menopause, as well as by informed innocent bystanders who would like to become better informed of the background and the latest developments in this medico-social conflict. In this book there is something for every one of those readers... and for some, there is a lot indeed.

The ethical and unethical conduct of some of the aforementioned stakeholders will not spare women the heavy decision of seeking professional help of whichever persuasion. In the end, the decision will necessarily be a narrowly personal one. The knowledge of the pros and cons should make such a decision a liberating exercise where there is no one position absolutely right. In some cases, this could be considered a Faustian pact aimed just at regaining youth, regardless of its ulterior medical health costs; in others, it may be just a sound therapeutic decision to improve unacceptable clinical symptoms. In still other cases, a combination of both extremes may make women decide to accept the medication and its risks. In any of these cases, knowing more will undoubtedly be advantageous to women who should make decisions with as much honest knowledge as possible

In several chapters, Professor Siegel Watkins proceeds to dissect who the targets were to the campaign to make estrogens and its combinations a "need" not an option. The author goes on to describe in great detail the strategy used by manufacturers to convince both doctors and patients of the benefits of using replacement therapy for a natural condition that used to be handled, admittedly at times painfully, without the risks of medication. In some chapters, the author clearly implies that well-regarded medical and scientific journals, as well as weekly magazines were somehow coaxed to publish articles or commentary that bent the truth regarding the merits and risks of HRT. From the 1940s to at least the 1970s such an unbiased peer-review process was ignored by editorial boards. The dissenters, and fortunately there were some even among lay publications, were not treated that well by the HRT advocates, and I'm using this word advisedly ("...I'm shocked!!!").

An interesting angle is explored by the author when she asks what has been the attitude of women at large to HRT. Publications have been available since the 1930s reporting that estrogens induced mammary cancer in surrogate laboratory models of carcinogenesis. Given the details generously referred to in this book, something (the pursuit of increasing shareholders value?) or someone (most probably, not the estrogen consumers) has chosen either to ignore or de-emphasize the ominous value of this evidence. This attitude is still well and alive today. For example, the compelling evidence collected by NIH-supported researchers should be taken as a warning about the consequences of lax regulation on the use of synthetic estrogenic compounds present in either consumables (beef, pesticide-tainted vegetables, etc) and plastic utensils that we come into contact with in our daily activities. As in the case of HRT, those who recommend restraint in the use of synthetic hormones to increase our "standard of living," have to rely on a few bench-scientists and on socially enlightened individual in the lay community who would speak up and concern themselves with popularizing to a rather large scientifically and socially poorly-educated audience,  the perils of exposure to these compounds.

So, yes, science and technology eventually correct themselves. But, at what costs to the innocent bystander who is unable to weigh the evidence favoring either side fairly? Starting in 1975 and following in subsequent years, the tide turned and estrogens began to show what the risks were in their indiscriminate use (increased incidence of endometrial and breast cancers). Thus, the results of the 1930s could have been extrapolated to those in the 1970s without the unnecessary (mostly anonymous) deaths and suffering of women who, misguided, took the wrong decision, subjecting themselves to HRT. The incidence of cardiovascular diseases also increased among HRT users.

In a society like ours where a culprit is supposed to be identified *a posteriori *for all things that go wrong (who lost China? Vietnam? Iraq?), this story is amenable to being treated in the same context. And the answer is... there is plenty of blame to be spread among all levels of a society that considers that we as consumers are here primarily to do business, and not to promote and enjoy societal understanding. An indication of the existence of this exceptionalism is backed up by statistics buttressing the finding that American women used estrogenic compounds for menopause-related effects in amounts much higher that those in Australia and Europe. Ultimately, a lack of social education in our affluent society might be the culprit; at the same time, the massiveness of those institutions involved is such that we all probably feel individually blameless.

If a criticism can be leveled at this book, it is that of being rather repetitious in presenting the testimony at hand. One may infer that in her responsible, scholarly effort, Professor Siegel Watkins wanted to be as informative and thorough as possible, thus providing the professional (and the personal) background and even some of the extracurricular activities of several of the protagonists. These, at times annoying, details however, detract much from the overall effective message of this cautionary tale. All in all, a comparable repetitious and regrettable technique has been used by the advocates of the HRT and, regrettably, with unchallengeable effectiveness. 

## Competing interests

The author declares that he has no competing interests.

